# Molecular basis of heterosis and related breeding strategies reveal its importance in vegetable breeding

**DOI:** 10.1038/s41438-021-00552-9

**Published:** 2021-06-01

**Authors:** Daoliang Yu, Xingfang Gu, Shengping Zhang, Shaoyun Dong, Han Miao, Kiros Gebretsadik, Kailiang Bo

**Affiliations:** 1 Institute of Vegetables and Flowers, Chinese Academy of Agricultural Sciences, Beijing, China; 2 Department of Plant Science, Aksum University, Shire Campus, Shire, Ethiopia

**Keywords:** Plant breeding, Gene regulation

## Abstract

Heterosis has historically been exploited in plants; however, its underlying genetic mechanisms and molecular basis remain elusive. In recent years, due to advances in molecular biotechnology at the genome, transcriptome, proteome, and epigenome levels, the study of heterosis in vegetables has made significant progress. Here, we present an extensive literature review on the genetic and epigenetic regulation of heterosis in vegetables. We summarize six hypotheses to explain the mechanism by which genes regulate heterosis, improve upon a possible model of heterosis that is triggered by epigenetics, and analyze previous studies on quantitative trait locus effects and gene actions related to heterosis based on analyses of differential gene expression in vegetables. We also discuss the contributions of yield-related traits, including flower, fruit, and plant architecture traits, during heterosis development in vegetables (e.g., cabbage, cucumber, and tomato). More importantly, we propose a comprehensive breeding strategy based on heterosis studies in vegetables and crop plants. The description of the strategy details how to obtain F_1_ hybrids that exhibit heterosis based on heterosis prediction, how to obtain elite lines based on molecular biotechnology, and how to maintain heterosis by diploid seed breeding and the selection of hybrid simulation lines that are suitable for heterosis research and utilization in vegetables. Finally, we briefly provide suggestions and perspectives on the role of heterosis in the future of vegetable breeding.

## Introduction

Heterosis occurs in a variety of species and has been observed and recorded in China since ancient times. For example, Jia Sixie described in *“The Manual of Important Arts for the People”* that interbreeding between horses and donkeys produced stronger mules, and the famous agricultural work *“Tian Gong Kai Wu”* also recorded crossbreeding techniques for silkworms. Heterosis has also been extensively studied in other countries. In 1763, the German scholar Koelreuter^1^ was the first to present concrete evidence that the growth of hybrid tobacco is superior to that of its parents. By comparing the height of hybrid and self-crossing offspring in maize, Darwin^2^ found that the average height of hybrid offspring was higher than that of self-crossing offspring. Beal^3^ found that the yield of maize hybrid offspring was greater than that of both parents. Shull^4,5^ observed heterosis in maize hybrid offspring and first proposed the concept of heterosis; he then formally named this phenomenon “heterosis.” Heterosis was first applied to genetic breeding in maize, and many excellent maize hybrids have been produced since the 1930s. Since 2011, the yield of maize increased by at least eightfold in America, due mostly to the cultivation of hybrids^6^.

As heterosis has been applied in cereal crop production, crossbreeding in vegetables has also rapidly progressed. Under natural planting conditions, 40–80% of seeds produced are usually hybrids due to fertilization competition between self-pollination and pollen from other plants^7^. Although the traits of randomly generated hybrid seeds are not organized at first, F_1_ hybrids exhibit higher yield, better adaptability, and higher stress resistance than pure line seeds under optimum production and fertilization protection management conditions. Therefore, farmers have paid much attention to the cultivation of hybrid seeds^8^. The first hybrid of eggplant (*Solanum melongena*) was released in 1924^9^. Subsequently, hybrids of other vegetables, such as watermelon (*Citrullus lanatus* L.), cucumber (*Cucumis sativus* L.), radish (*Raphanus sativus* L.), tomato (*Solanum lycopersicum* L.), and cabbage (*Brassica oleracea* L.), were developed over the next 20 years^7^. The number of hybrid vegetable varieties is rapidly increasing, at a rate of 8–10% each year, while nonhybrid vegetable varieties are gradually being eliminated^10^.

The application of heterosis to vegetable cultivation was first proposed by Hayes and Jones^11^ using cucumbers. However, because of the high cost of producing hybrid seeds, hybrid cucumber seeds were not used until the 1930s^7^. Similarly, self-pollination and the occasional presence of indehiscent anthers in eggplant^12^ and styles that are shorter than anthers in tomato^13^ have resulted in a high degree of self-pollination, which in turn has limited hybrid utilization. Pearson (1933) and Jones and Clarke (1943) used the mechanisms of self-incompatibility in cabbage and cytoplasmic male sterility in onion, respectively, to produce pure line and hybrid seeds on a large scale^8^. To avoid undesirable selfing, various genetic and nongenetic mechanisms, including genic male sterility, cytoplasmic male sterility, self-incompatibility, gynoecious lines, auxotrophy, and the use of sex regulators and chemical hybridizing agents, have been applied to facilitate hybrid seed production in vegetables^8,14^. The various traits that exhibit remarkable heterosis in F_1_ hybrids, including yield, earliness, growth vigor, and stress tolerance^15–18^, have become a major area of research on vegetables. In an experiment with hybrid eggplant conducted by Balwani et al.^19^ and Makani et al.^20^ heterosis in the optimal F_1_ hybrid resulted in yield increases of 125.78% and 88.88%, respectively. A more productive eggplant hybrid will effectively decrease the time to first harvest^18^. Transgressive phenotypes have also been observed in other Solanaceae^21,22^, Cruciferae^23,24^, and Cucurbitaceae vegetables^25,26^.

Although heterosis in vegetables has historically been used in research and crossbreeding experiments, its genetic mechanism remains elusive. Different genetic models for heterosis have been described in various reviews^27–31^. However, it is apparent that the classical genetic hypothesis of heterosis cannot explain all mechanisms of heterosis. Therefore, genetic models of heterosis have been included in this review. In addition to genetic models, we also present a schematic diagram depicting the involvement of epigenetics in heterosis. Simultaneously, we discuss studies on heterosis at the molecular level based on QTL effects and differential gene expression analyses. We also describe the effects of QTL on heterosis in crop plants based on Shang et al.^32^ to guide future research studies on the genetic mechanisms of heterosis. We summarize recent findings on the interactions of QTL sites with regard to heterosis and discuss the contribution of various QTL effects to heterosis. Differential expression analysis of genes related to heterosis can also provide a different perspective on heterosis^31^. In addition, we present morphological improvement as another measure to increase yield and an important component of breeding^7^ and describe how to combine heterosis utilization and morphological improvement.

To date, studies on heterosis in vegetables mainly involve obtaining F_1_ hybrids through crossbreeding. The utilization of cucumber hybrids proposed by Hayes and Jones^11^ was likely the first instance of effective vegetable breeding that exploits heterosis. Kumar et al.^30^ introduced methods of predicting heterosis in eggplant hybrids, such as genetic distance prediction and combining ability tests, and proposed the application of a sterile line system as well as transgenic and gene editing techniques in eggplant breeding. Herath et al.^33^ summarized the QTL mapping of yield-related traits in chili, introduced the use of heterosis breeding to improve the economic and agronomic traits of chili, and suggested the use of genomic technology and sterile line materials in chili breeding. Mallikarjunarao et al.^34^ reviewed the progress of various balsam pear (bitter gourd) hybridization tests and indicated that heterosis does occur in the yield of balsam pear hybrids. However, studies on the genetic mechanisms of heterosis in vegetables are limited, which hinders the application of heterosis in vegetable breeding. Therefore, in this review, we describe the progress of research on the genetic mechanisms of heterosis, analyze the use of hybrid production systems and molecular biology technology in vegetable production, and propose a breeding strategy that can predict, obtain, and maintain heterosis. This review will provide a reference for the utilization of heterosis in vegetable breeding.

## Study on the genetic mechanisms of heterosis

### Genetic regulation of heterosis

Heterosis is a complex biogenetic phenomenon caused by the combination of many factors that is manifested in the performance of hybrid offspring. The classical hypotheses for the genetic mechanisms of heterosis include the dominance and overdominance hypotheses, which are based on allelic interactions, and epistasis, which is based on nonallelic interactions.

**Fig. 1:**
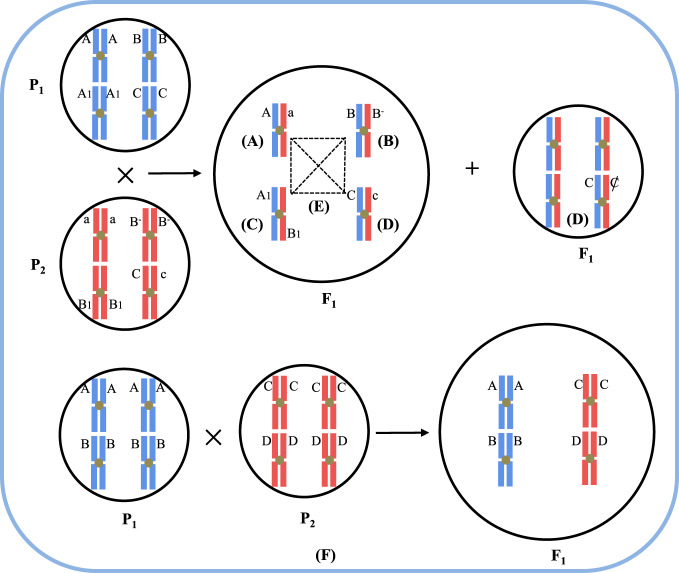
There are five hypotheses to explain the mechanism of heterosis based on gene effects. Suppose that the biomass is the sum of the genetic effects (A, B, C) and that the biomass of an organism is represented by the circular area. **A** Dominance effect: the dominant allele (**A**) inhibits the recessive allele (a); (**B**) overdominance effect: a single heterozygous allele (B/B^−^) promotes the development of heterosis; (**C**) Epistasis effect: nonallelic (A_1_/B_1_) interactions in the parents promote the development of heterosis; (**D**) active gene effect: genes from parents (**C**) promote heterosis when heterozygous and produce genome imprinting when homozygous, which inhibits the occurrence of heterosis; (**E**) gene network system: genes from parents (A, B, C) are combined into a coordinated gene network system that enables F_1_ to develop heterosis; (**F**) single-cross hybrids P_1_ (AB) and P_2_ (CD) produced from four homozygous inbred tetraploids (with genotypes A, B, C, and D) are crossed to produce F_1_ (ABCD), a double-cross tetraploid hybrid

Davenport^35^ first proposed the dominance hypothesis (Fig. 1A), and Bruce^36^ and Jones^37^ developed it further. In the dominant hypothesis, favorable genes controlling growth and development are dominant, and unfavorable genes are recessive. In the hybrid generation, the alleles from the two parents are complementary, and the unfavorable recessive genes are suppressed by the favorable dominant genes; therefore, the hybrid generation exhibits heterobeltiosis.

The overdominance hypothesis (Fig. 1B) was originally proposed by Shull^4^ and East^38^ as the opposite of the dominance hypothesis. This hypothesis denies that there is dominant-recessive relationship between alleles and suggests that the main cause of heterosis is the interaction of heterogeneous alleles from parents. Heterozygous alleles interact more strongly than homozygous alleles; thus, the hybrids exhibit heterobeltiosis. Using the isozyme technique, Dranginis^39^ found that the enzymes in heterozygotes exhibit many unique conformations of hybrid enzymes. For example, the regulatory proteins of heterozygotes often present as polymers that regulate genes, and different heterozygous and homozygous proteins consistently show different activity characteristics. In addition, the anthocyanin content heterobeltiosis that occurs due to the heterozygosity of a single locus (*pl*) in maize^40^ and the yield heterosis induced by the heterozygosity of a single locus (*sft*) in tomato^15^ also provide experimental evidence for the overdominance hypothesis. However, the interaction of closely linked alleles can also result in an overdominance effect that is known as pseudo-overdominance^41^.

The dominance and overdominance hypotheses for the heterosis phenomenon both suggest that heterosis is caused by individual allele loci. However, several reports have shown that plant traits such as yield and growth vigor are complex quantitative traits^42^. Wright^43^ visualized the network structure of population genotypes, i.e., multiple loci control the variations in most traits; in such networks, the replacement of anu gene may affect multiple traits. Based on this perspective, Sheridan^44^ proposed the concept of epistasis. He believed that heterosis may arise from interactions between nonalleles. In genetics, the phenomenon in which the genetic effect of a nonallele deviates from its additive effect is called epistasis (Fig. 1C). The significant special combining ability (SCA) effects in the hybridization experiment of Sao and Mehta indicated that epistasis plays a predominant role in the genetic control of eggplant heterosis^45^. Using a genetic map that covered the whole rice (*Oryza sativa*) genome, QTL mapping for yield-related traits was conducted in 250 F_2:3_ lines. The results showed that the correlation between marker heterozygosity and yield-related traits was low and that the interaction between most genes could not be detected on the basis of single-gene loci; the interactions were classified as dominance by dominance, additive by dominance, and additive by additive^46^. Therefore, Yu et al.^46^ also believed that epistasis is an important genetic basis for the development of heterosis.

Other ideas in addition to the classical hypotheses have been proposed. Zhong^47^ proposed the active gene effect hypothesis (Fig. 1D) by comparing the relationship between genomic imprinting and heterosis; this hypothesis suggests that heterosis is caused by additive effects between the active genes. When alleles are homozygous, only one of them is active. When genes are heterozygous, genomic imprinting does not occur, and all genes are active, showing all effects. The interaction between active genes increases the overall effect of gene expression; as a result, the hybrid exhibits heterosis. For example, in maize, the red1 (*r1*) gene, when inherited from both parents, causes different colors in corn kernels^48^. Genomic imprinting affects the differential expression of genes by affecting DNA methylation and histone modification^49^. Bao^50^ suggested that individuals have a specific set of genetic information that controls their growth. Genetic information is expressed as different coding genes in organisms; these genes form an orderly network of expression, and the activities of each gene are related to each other. An alteration in a single gene may cause changes in the entire network. The network of F_1_ hybrids is a new gene network system that is formed from the two different gene networks of the parents. If the interactions between alleles bring the whole genetic network system to an optimal state, the F_1_ hybrid exhibits heterosis; otherwise, it remains typical (Fig. 1E). In addition, the effects caused by genomic imprinting or active gene effects may be components of genomic dosage effects^51^; the other part of genomic dosage effects usually caused by polyploidy, which is a specific phenomenon in polyploid plants called progressive heterosis (Fig. 1F)^52,53^. The genomic dosage effects produced by allopolyploids are usually stronger than those produced by homologous polyploids^38,51,54,55^. The formation of polyploids is accompanied by extensive genetic and epigenetic changes^56^, which may provide the molecular basis for the development of heterosis.

### Epigenetics is involved in the development of heterosis

Although many hypotheses have been proposed to explain the mechanisms of plant heterosis at the genetic level, studies have shown that the genetic mechanisms of heterosis cannot be fully explained by one or even several hypotheses at the genetic level. Through the intensive study of epigenetics, epigenetic factors such as DNA methylation, small RNAs, and histone modifications have been found to be involved in the development of heterosis in plants^57–62^.

**Fig. 2:**
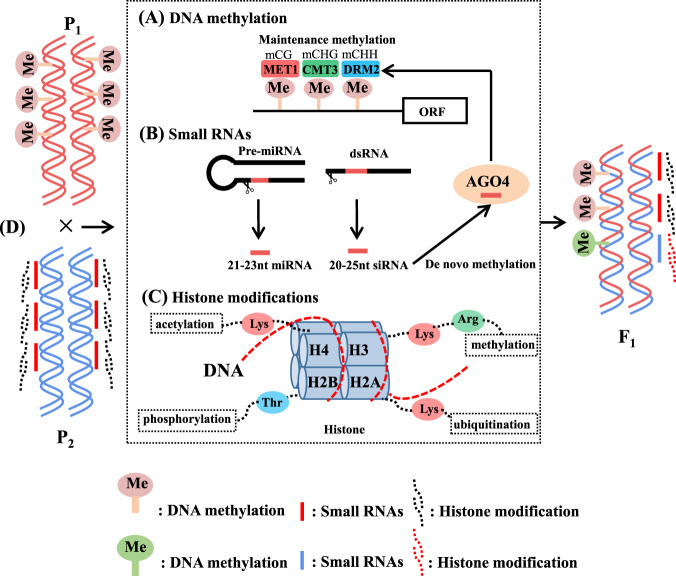
Putative model of heterosis triggered by epigenetics. **A** DNA methylation: De novo methylation was catalyzed by DRM2, a homologous enzyme of DNMT3. In maintenance methylation, CG is catalyzed by MET1, a homologous enzyme of DNMT1; CHG is catalyzed by CMT3; and CHH is still catalyzed by DRM2. **B** Small RNA: Includes the miRNA produced by premiRNA and the siRNA produced by dsRNA. In general, 24 nt-siRNA mediates de novo DNA methylation catalyzed by the AGO4 protein. **C** Histone modifications: The modifications of histone amino acid residue includes acetylation, phosphorylation, methylation, and ubiquitination processes. Epigenetic modifications are produced by the parents. New epigenetic modifications may occur in F_1_ hybrids. **D** Epigenetic modification status of the parents and F_1_ hybrid: the increase and decrease in or recombination of epigenetic modifications induces the F_1_ hybrid to exhibit heterosis

Epigenetic modifications play an important role in the formation of plant phenotypes by regulating gene transcription and gene expression^63–65^. Alleles of known phenotypes have been studied more extensively in the context of DNA methylation than in the context of other epigenetic modifications^63^. RNA-directed de novo methylation (RdDM) is one of the pathways that triggers DNA methylation by 24 nt-siRNA, which is regulated by two key genes, namely, *NRPD1* and *NRPE1*^66^ (Fig. 2A, B). A silent epigenetic variant caused by differentially methylated regions (DMRs) in the promoter, *sulfurea* (*sulf/+*), can result in homozygous lethal tomato plants that exhibit only chlorotic leaf sectors^64,65^. This may occur due to the random combination of genetic information from the parents of the F_1_ hybrids because their genotypes are more prone to heterozygosity at the DNA methylation level; this is in line with the findings of Shen et al.^59^. The gene effect caused by such heterozygosity may enable F_1_ hybrids to avoid producing common phenotypes or hybrid weakness, thus achieving heterobeltiosis. Using experiments involving heterograft eggplants, Cerruti et al.^62^ found that scion vigor is related to DNA methylation and that the reduction in methylation in the CHH context promotes scion vigor. Tomato grafting experiments revealed that RdDM can cause a heritable enhancement-through-grafting phenotype^67,68^.

Because de novo DNA methylation is mediated by siRNAs (Fig. 2B), siRNAs may also be involved in the regulation of heterosis. The level of siRNAs decreased in different genome regions between parents and hybrids, but this phenomenon was limited to 24 nt-siRNAs; in contrast, the levels of siRNAs of other sizes did not decrease^67^. Noncoding small RNAs can be used as signaling molecules in plants^67^. Shivaprasad et al.^61^ observed that miR395 is differentially expressed, mediates transgressive phenotypes in the hybrid progeny of tomato and is associated with suppression of the corresponding target genes, which indicates that the combination of parental genetic information can cause differences in miR395 abundance in the progeny. Simultaneously, 21–24 nt small RNAs can move through the intercellular filaments and phloem of the graft site^69^, and 24 nt sRNAs can guide genomic DNA methylation in recipient cells^70^; this information provides a theoretical basis for guiding grafting. In addition, sRNAs in plants usually play a major role in inducing gene expression silencing and gene posttranscriptional silencing^71,72^. This may be due to the downregulation of sRNA levels in hybrids, which lifts the silencing of some favorable genes and thus allows hybrids to exhibit heterobeltiosis^71,72^.

Different modifications, such as acetylation, phosphorylation, methylation, and ubiquitination, occur at the amino terminus of histones (Fig. 2C). These histone modifications can affect the binding of related proteins to chromatin and thereby affect the transcriptional activity of genes. At the same time, the combination of modifications of the amino terminus of histones expands the genetic information for and changes the phenotype of an individual^73^. Histone modifications are related to the stability of heterosis. Studies have shown that histone deacetylases cause the nonadditive expression of some genes in hybrids^58^. In addition, histone acetylation and methylation are related to the activation of regulatory (circadian-regulated) genes in F_1_ hybrids^73^. The biological clock controls the physiological activities of plants, including the synthesis of physiological and biochemical substances. Therefore, histone modifications can influence plant biomass heterosis.

The recombination of genetic information from parents may lead to new combinations of epigenetic modifications in the F_1_ generation (Fig. 2D). Epigenetic modifications essentially affect the expression of genes, causing them to be overexpressed or silenced. Therefore, epigenetic modifications may indirectly influence the development of heterosis in F_1_ by affecting the expression pattern of genes.

## Study on heterosis at the molecular level

### Progress in heterosis research based on QTL analysis

**Fig. 3:**
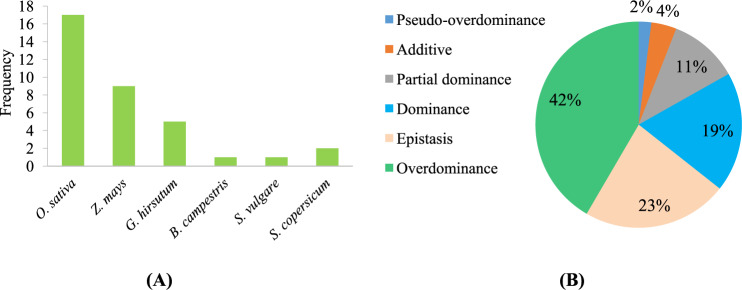
**Statistical analysis of the effect of quantitative trait loci on crop heterosis. A** In the statistical analysis of the effect of quantitative trait loci on crop heterosis, the species and frequency of each species were studied; (**B**) in the statistical analysis of the effect of quantitative trait loci on crop heterosis, the quantitative trait locus effect on each species and the proportion of each type of effect were analyzed

The genome contains all the genetic information of a species and determines whether an individual gene is expressed as well as its degree of expression. Heterosis is usually indicated if the hybrid generation is superior to the parents in terms of quantitative traits. Thus, it is essential to conduct a genetic analysis of heterosis from the perspective of the whole genome. With the rapid development of genome sequencing technology, it has become possible to identify gene loci related to heterosis by genome-wide association studies^74^, which lay a foundation for the study of individual phenotypic differences. This review summarizes the QTL effects on heterosis based on 35 studies that mainly addressed 6 crops and vegetables, i.e., rice (*Oryza sativa*), maize (*Zea mays*), cotton (*Gossypium hirsutum*), oilseed rape (*Brassica campestris*), sorghum (*Sorghum vulgare*), and tomato (*Solanum lycopersicum*) (Table S1). Among the six types of QTL effects, dominance and epistasis had equal proportions (19%, 23%, Fig. 3). Interestingly, the overdominance effect accounted for the largest proportion of all the effects (42%, Fig. 3). This means that although there are many gene loci in the plant genome, these interacted to produce different, complex, hard-to-imitate effects and resulted in heterosis; among these effects, overdominance effects occurred consistently and contributed significantly to heterosis. In addition, the overdominance effect can be conveniently used for artificial breeding, which has been well demonstrated in tomato^15^. However, efficiently and accurately locating the gene loci that impart the overdominance effect is necessary to make use of this effect. Heterosis may be the result of many traits. In addition, the results of QTL mapping differ among species and even within different groups of the same species^75–77^. Therefore, it is necessary to select a suitable genetic population based on the genetic background of the plants exhibiting heterosis.

### Advances in gene action related to heterosis based on differential expression analysis of genes

The genome controls the formation of a biological phenotype by regulating the differential expression of genes^78,79^. Molecular-based expression analyses, such as allele-specific expression, DNA microarray, expression quantitative trait loci, RNA-seq, quantitative SNP-based Sequenom technology, and allele-specific RT-PCR, have made it possible to detect differential gene expression.

**Fig. 4:**
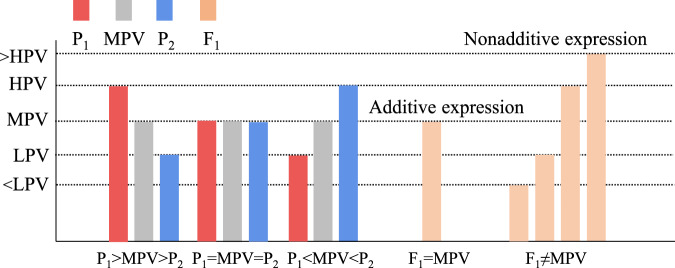
By comparing the gene expression of the F_1_ hybrid and its parents, the gene expression patterns of F_1_ were divided into additive gene expression patterns and nonadditive gene expression patterns. Midparent value [MPV = (HPV + LPV)/2]; High-parent value (HPV); low-parent value (LPV)

Yield and biomass heterosis in F_1_ hybrids may occur due to the altered expression patterns of genes that control biological functions such as carbon fixation, glucose metabolism, and circadian rhythm^80^. Gene Ontology (GO) analysis of pakchoi line parents and hybrids indicated that most of the differentially expressed genes between parents and hybrids enriched the photosynthetic pathway and that the enhancement of the photosynthetic capacity of the hybrids was related mainly to an increase in the number of thylakoids^17^. In addition, the increase in the number of thylakoids also promoted the enhancement of the carbon fixation capacity in the hybrids^17^; this is similar to the finding that differentially expressed genes that significantly enrich the optical signaling pathway occur between F_1_ and their parents in broccoli^24^. The same results were also found in other plants^79,81^. Transcriptome and differential gene expression analyses revealed that the modes of action of heterosis genes were mainly additive (F_1_ = MPV), overdominance (F_1_ > HPV), and underdominance (F_1_ < LPV)^82^ (Fig. 4). When the expression value of a differentially expressed gene in the hybrid line was higher or lower than that of the parent, the gene action patterns were classified as high-parent dominance (F_1_ ≈ HPV) and low-parent dominance (F_1_ ≈ LPV), respectively^82^ (Fig. 4). Li et al.^24^ reported that most genes exhibited additive expression patterns in hybrid broccoli and that nonadditive action was involved mainly in light and hormone signal pathways related to heterosis; a similar finding was reported in Chinese cabbage (*Brassica campestris* ssp. *pekinensis* cv. “*spring flavor*”)^23^. These gene expression patterns may have occurred due to selective inhibition or activation by the epigenetic modification of hybrid F_1_ genes^83,84^; the genes from inactive inbred lines can be activated by genes or regulatory factors of active inbred lines^85,86^. Epigenetic modifications and the interactions of heterogeneous factors occur in only a few genes, and the genome that produces differential expression in F_1_ hybrids and parents accounts for only a small part of the total genome^87^. Moreover, Springer and Stupar^88^ have shown that additive gene expression accounts for the majority of gene expression, while nonadditive gene expression is responsible for a small proportion of gene expression. These findings suggest that nonadditive expression of this fraction facilitates the development of heterosis.

## Traits contributing to yield heterosis in vegetables

### Traits related to yield heterosis

**Fig. 5:**
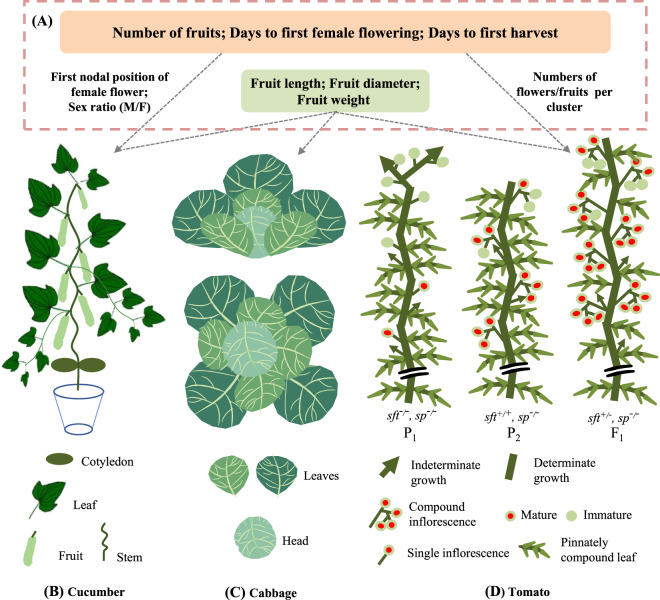
**Contributing traits of yield heterosis in cucumber, cabbage and tomato. A** Traits contributing to yield heterosis in cucumber, cabbage, and tomato: cucumber yield contributing traits include the number of fruits, days to first female flowering, days to first harvest, first nodal position of female flower, sex ratio (M/F), fruit length, fruit diameter, and fruit weight; cabbage yield contributing traits include fruit length, fruit diameter, and fruit weight; tomato yield contributing traits include number of fruits, days to first female flowering, days to first harvest, number of flowers/fruits per cluster, fruit length, fruit diameter, and fruit weight. **B** Cucumber: cucumber model in production, gynoecious line with a small number of branches. **C** Cabbage: an aerial and cross-sectional model of cabbage consisting of leaves and heads. **D** Tomato: a tomato with single inflorescences and indeterminate growth is crossbred with a tomato with compound inflorescences and determinate growth to produce the hybrid F_1_ with earlier fruiting, more compound inflorescences, and determinate growth

Hybrids that exhibit heterosis show significant heterobeltiosis in yield, which is a complex trait that is usually measured by weight. To clearly study the mechanisms of yield increase in hybrids, it is essential to divide yield into other, simpler traits. This review describes the traits that contribute to vegetable yields. Fruits are the source of the yield of most plants; the yield contributing traits related to fruits usually include the fruit number, fruit size and fruit weight; earliness is usually also taken into account. Cabbage is a typical leafy, head-forming vegetable in Cruciferae, so its main yield contributing traits are head weight and head size (Fig. 5A, C). Similar to that of cabbage, the yield of radish is determined by its taproot. For leafy vegetables that do not form heads, the main yield heading traits are the number and size of the leaves. Unlike cruciferous vegetables, Cucurbitaceae and Solanaceae vegetables are produce multiple harvests and multiple fruits per plant (Fig. 5B, D), so the average single fruit weight and fruit yield per plant should be taken into account. In addition, Solanaceae vegetable flowers consist mostly of compound inflorescences^89^, so the numbers of flowers per cluster and fruits per cluster contribute greatly to production. Cucurbitaceae are single-inflorescence vegetables; only the fruits on the main vine are harvested in production, and the first nodal position of female flowers and sex ratio (M/F) affect the days to first harvest and the number of fruits per plant, respectively. Regardless of the trait considered, the total yield can be affected only by changes in yield-related traits. Therefore, it is necessary to analyze the mechanisms that regulate yield-related traits.

### Relationship between yield heterosis and plant architecture

Since the “green revolution”, interest in breeding for specific plant architecture has significantly increased, and the idea of combining heterosis breeding with plant architecture breeding has been proposed^90^. Donald^91^ conducted research on half-dwarf plant architecture, which gradually turned into the concept of the ideotype. Donald introduced the ideotype concept, which refers to the plant architecture form that results in the minimum competitive intensity in population breeding. Although this definition is no longer used, the concept of an ideal plant architecture has played a major role in promoting plant breeding for high yields. Research on ideotypes first made progress in rice. It is worth mentioning that a key gene regulating ideotype, *IPA1*, was proven by Huang et al.^75^ to influence genes that are important in heterosis by using the indica-japonica hybrid rice group. Studies of heterozygosity and ideotype were also combined effectively in tomato. The self-pruning (*sp*) gene promotes indeterminate growth in tomato, while the *sft* gene changes indeterminate growth into determinate growth by inhibiting the *sp* gene^92^. The *sft* gene results in the development of heterosis in tomatoes through the heterozygosity of a single gene^15^ and induces changes in plant architecture on the ground, causing tomato to produce compound inflorescences rather than single inflorescences^93^. The earliness of F_1_ was also higher than that of its parent (Fig. 5D), which increased tomato yield. Other vegetables in addition to tomato may also have ideotypes, and the key genes controlling plant architecture may also be important genes that are involved in the development of heterosis. Therefore, it is particularly important to study the genetic mechanisms of heterosis. By identifying the important genes involved in heterosis, the key genes that control plant ideotypes can be characterized.

### Advances in heterosis utilization and biotechnology in vegetables

Breeding for heterosis has been extensively studied in plants, and research on the heterobeltiosis of hybrid offspring in vegetables has focused mainly on yield^94^ and disease resistance^29^. Wellington^95^ and Tschermak^96^ showed that tomato hybrids exhibit heterosis in early maturity and during yield production. Krieger et al.^15^ cloned the single-gene *sft* that affects the female flower fertility rate in tomato by infiltrating the IL and TC populations. When the *sft* gene exhibited heterozygosity, the tomato yield exhibited heterosis. According to this study, tomato plants that showed yield heterosis also showed resistance to both biological and abiotic stresses. The heterozygous state of the *Tm* and *Tm22* genes contributes to tobacco mosaic virus resistance^97,98^ and high-temperature stress tolerance^99,100^. Naresh et al.^101^ suggested that heterosis is the result of nonadditive gene effects and that it also plays an important role in improving Cercospora leaf spot resistance in eggplant in the field. Similar to studies on other vegetables, studies on heterosis in Cucurbitaceae vegetables have also focused mainly on yield and disease resistance. Pandey et al.^102^ used 77 cucumber hybrid generations and their parents to study the yield heterosis and contributing traits of different cucumber hybrid varieties and found that DC–1 × B–159 and VRC–11–2 × Bihar–10 were the best hybrid combinations for yield and prematurity. Using 48 F_1_ hybrids and their parents, the gene effects caused by diseases and insect pests under natural conditions^29^ were investigated. The results indicated that nonadditive gene effects had a significant regulatory effect on other traits in cucumber (except morbidity caused by Drosophila), demonstrating the importance of heterosis in cucumber breeding for disease resistance.

Different molecular markers, such as simple sequence repeats (SSRs), inter-simple sequence repeats (ISSRs), amplified fragment length polymorphisms (AFLPs), random amplified polymorphic DNAs (RAPDs), and sequence-related amplified polymorphisms (SRAPS), have provided the molecular basis for the construction of genetic maps and the mapping of important trait genes (Table 1). Whole-genome sequencing has been conducted for a variety of vegetables (Table 1), which has provided a basis for whole-genome strategies. Whole-genome approaches can help obtain complete sequences of germplasm resources, increase the coverage of molecular markers, and increase the accuracy of genetic maps^103^. Molecular markers are often used for the determination of genetic distance and the classification of heterotic groups. To elucidate the breeding processes and to improve the efficiency of breeding techniques in cabbage, heterotic cabbages are usually divided into two groups: The round head type and the flat head type. Xing et al.^104^ further divided 21 flat cabbage inbred lines into three heterotic groups and divided 42 round cabbage inbred lines into five heterotic groups in order to provide a more definite direction for the preparation of hybrid combinations of cabbage. The method of dividing heterotic groups by molecular markers and genetic distance is widely used in vegetable breeding (Table 1).

**Table 1:** Research progress in vegetable breeding

Vegetable variety	Identification of heterotic groups	Maintenance of heterozygosity during production	Whole-genome sequencing	Regeneration system
Chinese cabbage	SSR, GD, ISSR, genealogical information	Polima CMS (*MsMs*)	Yes (Wang et al.^186^)	Cotyledon-petioles (Li et al.^187^)
Cabbage	SSR, microsatellites	TGMS (*Ms*); PGMS	Yes (Liu et al.^188^)	Hypocotyl (Ma et al.^189^)
Radish	AFLP, ISSR, SSR	Male sterility; self-incompatibility	Yes (Kitashiba et al.^190^)	Microspore (Kim et al.^191^)
Tomato	RAPD, Indel, SSR, AFLP, SNP	TGMS: Sterile pollen (*ms*); lack of stamens (*sl–1, sl–2*); positional sterility (*ps*); functional sterility (*ps–2*)	Yes (Consortium^192^)	Hypocotyl and cotyledon (Lee et al.^193^)
Potato	GD, SNP, RAPD	Vegetative propagation	Yes (Consortium^194^)	Stem segments (Zhang et al.^195^)
Pepper	GD, AFLP, SSR, RAPD	TGMS (*ms–10, ms–12*); TCMS	Yes (Kim et al. 2014; Qin et al.^196,197^)	Hypocotyl (Chee et al.^198^)
Eggplant	GD, AFLP, SSR, combining ability test	GMS; functional male sterility (*fms*)	Yes (Hirakawa et al.^199^)	Leaf (Khan^200^)
Cucumber	SSR, GD, AFLP, combining ability test	GMS (*ms–3*); gynoecious	Yes (Huang et al.^201^)	Cotyledon (Du et al.^202^)
Melon	SSR, GD, combining ability test	GMS (*ms–3, ms–4, ms–5*); gynoecious	Yes (Garcia–Mas et al.^203^)	Cotyledon (Chenarani et al.^204^)
Watermelon	RAPD, SSR	GMS (*gms*); gynoecious	Yes (Guo et al.^205^)	Cotyledon (Vasudevan et al.^206^)
Pumpkin	RAPD, SRAP	Gynoecious	Yes (Montero–Pau et al.; Sun et al.^207,208^)	Cotyledon (Guo et al.^209^)

Chen^83^ proposed that determining how to obtain hybrid seeds is the key to the utilization of heterosis. The purpose of obtaining hybrid seeds is to make heterosis in the offspring permanent. The sporophyte of cruciferous vegetables is a self-incompatible system^105^ that can prevent self-pollination and produce normal seeds through cross-pollination. Hence, this system is convenient for the generation of hybrid seeds. In cabbage^106,107^ and Chinese cabbage^108^, hybrids are usually obtained using self-incompatible and male-sterile lines. To produce hybrid tomato seeds, pollen-abortive type and functionally sterile lines are often used^109–111^. Cytoplasmic male sterility occurs in eggplant^112,113^ and pepper^114,115^. Gynoecious lines tend to exist in Cucurbitaceae^116^. A new male-sterile system in tomato was developed by Du et al.^117^. Plant growth regulators such as ethylene, auxins, and brassinosteroids^118,119^ can increase the number of female flowers in Cucurbitaceae; this effect and male sterility are both convenient for hybrid seed production.

## Strategies for heterosis breeding in vegetables (with tomato as an example)

### Obtaining F_1_ hybrids that exhibit heterosis based on heterosis prediction

**Fig. 6:**
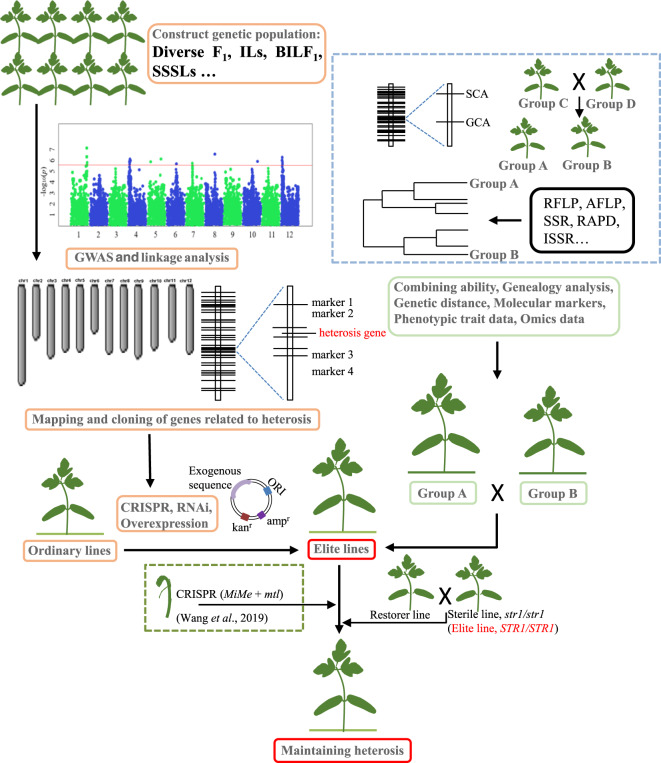
There are two key factors involved in applying heterosis breeding strategies: obtaining heterotic lines and maintaining heterosis in the elite lines in the offspring. There are two strategies for obtaining heterotic lines in crop breeding. The first is the use of crossbreeding or molecular biotechnology. Genealogical analysis, molecular markers, combining ability, and genetic distance can usually predict heterosis development, so they are often used to classify heterotic groups. The inbred lines from different heterotic groups can be crossed with each other to obtain elite lines that exhibit heterosis. The second strategy is to use modern molecular biotechnology. Elite lines were obtained based on GWAS and linkage analysis, mapping and cloning genes related to heterosis, gene editing, and gene transformation

It is not advisable to conduct extensive hybridization tests to obtain hybrid F_1_ lines that exhibit heterosis, as this approach requires considerable resources and time and produces unreliable results^13^. Melchinger and Gumber^120^ proposed that heterotic groups should be used as the basis for crossbreeding. The heterotic group is the population that is classified according to breeding requirements, with abundant genetic variation and high combining ability. Chen et al.^121^ carried out a genome-wide association study (GWAS) on the yield traits, general combining ability (GCA), and SCA of rice. The study provided strong evidence for the use of combining ability to classify heterotic groups and provided a reference for studies on combining ability in vegetables (Fig. 6). Other studies have also shown that combining ability, genetic distance, and molecular markers can provide the basis for evaluating parental inbred lines and predicting F_1_ hybrid heterosis in vegetables^122–125^.

The GCA characterizes the average performance of a set of hybrid combinations and is mainly the consequence of additive gene effects and additive × additive interactions; SCA evaluates the average performance of certain hybrid combinations compared to the parental lines and is the result of dominance, epistatic deviation and genotype × environmental interactions^126^. Parents with a high GCA effect have higher adaptability and fewer environmental effects^127^. Parents with superior traits do not always pass on their traits to offspring^126^; hence, the evaluation of combining ability is more reliable than the performance of the lines per se. Many types of combining ability tests can be used to identify superior parental lines for developing heterotic hybrids, including line × tester analysis, topcross tests, single-cross tests, poly-cross tests, and diallel mating^128^. Singh et al.^129^ conducted a complete diallel cross test on seven diverse bitter gourd lines and found that combinations with high × high GCA usually produced high SCA effects and could therefore be considered for use in developing superior variants through the pedigree method. High/low × low GCA combinations can also achieve high but unstable SCA effects that are suitable for heterosis breeding and are in line with the results of Kenga et al.^130^ in sweet sorghum and Franco et al.^131^ in common bean.

In addition to combining ability, heterotic groups are often classified by genealogical information^132^. For parents with known genealogical relationships, heterosis in hybrids can usually be predicted according to these genealogical relationships. Genetic distance is a quantitative description of the genetic differences that provide the genetic basis for the development of heterosis in offspring^133,134^. Parental lines with a longer genetic distance are more likely to produce hybrids with strong predominance^135,136^. Molecular markers can also be used to directly or indirectly classify heterotic groups by assessing their genetic distance^125,137,138^. RAPD and AFLP have been successfully used to detect the genetic distance between tested lines, and the yield of carrots was found to be significantly correlated with genetic distance^125^. Genetic distance has also been applied to predict hybrid pepper fruit diameter^139^ and hybrid melon (*Cucumis melo* L.) fruit shape diameter^140^. The scientific classification of heterotic groups improves the efficiency of selecting hybrid combinations of superior parents and utilizing heterosis (Fig. 6).

In addition, some omics approaches, such as genomics, transcriptomics, and metabolomics, have become tools for predicting hybrid yield in rice^141^. Xu et al.^141^ analyzed metabolomic and genomic data from 21,445 hybrids developed by 210 recombinant inbred lines and found that metabolomic data were more effective than genomic data in predicting hybrid yield. Research on the prediction of heterosis in vegetables with omics data has not been published. However, the genome or epigenome is the most fundamental source of the plant phenotype, and the transcriptome, proteome, and metabolism are the direct sources of plant phenotypes. Therefore, omics data could represent a more accurate way to predict vegetable hybrid heterosis, and studies of crop hybrid yields can provide a reference for predicting heterosis in vegetables.

### Obtaining elite lines based on molecular biotechnology

GWAS is a method used to identify the gene loci that control certain traits in a population by combining phenotypes with genotypes. GWAS is often used to identify certain traits, such as green flesh color or thermotolerance, in cucumber^142,143^ but can also be used to analyze complex traits, such as yield and biomass^144–156^. In addition, whole-genome sequencing of various vegetables provides a basis for GWAS (Table 1). Due to the unique phenotype of heterosis and its genetic background sources, a genetic population can be composed of different populations or ecotype hybrid populations. A segregated F_2_ population that was produced by a strongly predominant F_1_ population is regarded as the best population for studying heterosis^27^. Such an F_2_ population not only has a reasonable proportion of lines with heterozygous genotypes and homozygous genotypes but also has allele combinations that are distributed evenly at each site^27^.

DeVicente and Tanksley^157^ randomly paired an RIL population obtained by strong F_1_ self-crossing to produce a new population. This population not only preserves the genotype of the RIL population but also reproduces the F_2_ population; thus, it is called an IF_2_ population. At present, IF_2_ populations have been established in rice^158–161^, maize^150,162–169^, cotton^170^_,_ and other crops. In addition, there are also diverse F_1_^156^, IL^171–175^, BILF_1_^176,177^, and SSSL^178^ populations that can be used to study heterosis. Except for two studies on tomato, there are few relevant studies on heterosis in vegetables using such populations that would provide a reference for conducting heterosis-related studies in other vegetables.

Using genome editing techniques to knockout adverse genes or overexpress favorable genes can transform ordinary lines into strong predominance lines. For example, biomass, plant height, and leaf photosynthetic pigment contents increased in rice expressing maize *GLK* genes compared with those in wild-type rice;^179^ such results may cause researchers to think about studying mutual heterosis promotion among different vegetables. Dominance and overdominance effects account for a large proportion of the effects that produce heterosis and are easy to mimic (Fig. 3B). Understanding the mechanisms of heterosis helps breeders to improve current varieties and generate novel cultivars^27^ (Fig. 6).

### Maintaining heterosis

The hybridization of the selfing line of two heterotic groups can generate hybrid offspring that exhibit heterosis. Through hybrid seed production, self-incompatibility and male-sterile line technology can be used to maintain the hybrid vigor of the hybrid F_1_ line. Some of the characteristics of the vegetables themselves, such as the gynoecious characteristic of Cucurbitaceae^116^ and asexual reproduction in potato (*Solanum tuberosum* L.)^180^, are convenient for hybrid seed production or heterosis maintenance. In addition, some plant hormones or chemical reagents can also be used for plant sex regulation^14^. However, exogenous regulation is often not completely effective^14^, which may affect the purity of hybrid seeds. Therefore, it is necessary to study hybrid systems of vegetables for hybrid seed production.

Du et al.^117^ used gene editing technology (Cas9) to knock out the male-specific gene *SlSTR1* in tomato to obtain a sterile line and generated a maintainer line by transferring a fertility-restoration gene to the sterile line; it was easy to distinguish whether offspring of crosses between the maintainer and male-sterile lines were male-fertile maintainer plants because a seedling-color gene was linked to the fertility-restoration gene. This system combined tomato sterile lines and gene editing technology and represents a highly practical potential approach to hybrid seed production in tomatoes. Moreover, it may serve as an important reference for the use of gene editing technology for hybrid seed production in other vegetables.

Khanday et al.^181^ and Wang et al.^182^ found that genome editing can cause mitosis to replace meiosis in rice such that diploid clonal seeds have the original F_1_ gene heterozygosity and maintain F_1_ traits (Fig. 6). Unlike with knocking out the infertility gene using gene editing technology, with this method, fertilization and cell division are necessary for hybridization. Some vegetables do not have sterile line material. Therefore, this method, in which plant fertilization involves only mitosis and not meiosis, will be more widely applicable.

In addition, by repeatedly screening the F_2_ lines that were close to the F_1_ phenotype, Wang et al.^85^ obtained pure F_5_/F_6_ lines that were close to the F_1_ phenotype; these were called hybrid simulation lines, indicating that the phenotype of the F_1_ hybrids was fixed in this line. This method has also been used to maintain F_1_ heterosis in other vegetables, such as tomatoes^183^ and peas (*Pisum sativum* L.)^184^. Therefore, the heterosis of hybrid F_1_ vegetables produced by hybridization or molecular biotechnology can be maintained by diploid seed breeding and selection for hybrid simulation lines in the future (Fig. 6).

## Conclusions and future perspectives

Research on vegetable heterosis has focused mainly on its applications in heterosis breeding. Studies on its genetic mechanism are limited, which hinders its utilization. Extensive progress has been made in the study of heterosis in cereal crops such as rice and maize. In vegetables, both hybrid production systems (male sterility lines, self-incompatibility lines, and gynoecious lines) and molecular biological techniques (gene editing, transgenosis, and asexual reproduction) have been used. Therefore, the methods and strategies proposed by this paper for studying the genetic mechanisms of heterosis can be applied to vegetable breeding. In the near future, we will identify certain heterosis-related gene loci in vegetables to understand the molecular genetics and mechanism of heterosis formation in vegetables and to make new breakthroughs in improving the yield, quality, and safety of vegetables. This review emphasizes the following points: (1) The application of heterosis in vegetable crops allows improvements in yield and quality and enhances plant resistance to biological and environmental stresses. (2) In the future, more attention should be paid to the study of the genetic mechanisms of vegetable heterosis to identify the important genes involved in the development of heterosis and to understand the regulation and activity modes of the key genes affecting vegetable heterosis. (3) By fully referencing and adapting the strategies used in cereal crop heterosis studies, exogenous genes can be applied to produce the same function in different species^179^. Therefore, transgenic and genomic editing technologies can significantly improve the efficiency of research on heterosis gene identification in vegetables. (4) Although a certain basic molecular knowledge of vegetable heterosis has been obtained, applying the knowledge acquired from cereal crops to vegetables will improve vegetable production and quality. It will also be useful to compare sterile line seed production with optimized transgenic systems to achieve more breakthroughs in vegetable production. (5) The study of heterosis can promote the study of ideal plant architecture in vegetable breeding. A breeding strategy that combines heterosis with the ideal plant architecture can achieve substantial gains in vegetable yield and quality. (6) Maintaining heterosis is the core factor of the extensive use of heterosis and has been reflected mainly in F_1_ hybrid seed production. With the development of gene editing technology, sterile line gene editing systems, *MiMe* (Cas9) systems and even new biotechnology approaches will have opportunities to be widely applied; this will be of great significance for hybrid seed production. (7) Progressive heterosis caused by the dosage effect in polyploid hybrids is also an important component of the genetic mechanisms of heterosis, and these phenomena have been observed in different plants^55,185^. Polyploid systems allow experiments to be performed that are impossible in diploid systems; hence, polyploid crossbreeding may lead to different plant performance results than diploid breeding. However, polyploids have highly heterozygous genomes and complex genetic structures, and we may not be able to evaluate their phenotypes and genetic structures using diploid criteria. This topic deserves future investigation.

## Supplementary information

Supplymental Table 1

## References

[1] Koelreuter, J. In *Methods of Plant Breeding* (eds. Hayes, H. K. & Immer, F. R. & Smith, B. C.) (Mcgraw Hill Book Co. Inc., 1763).

[2] Darwin, C. *The Effects of Cross and Self Fertilisation in the Vegetable Kingdom* (D. Appleton, 1885).

[3] Beal, W. Re. Michigan Board Agric. P 287-288. Cited in Hallauer, AR and J. B. Miranda (1988). Quantitative genetics in maize breeding. (IOWA State Univ. Press Ames, 1880).

[4] Shull, G. H. The composition of a field of maize. *J. Hered*. **4**, 296–301 (1908).

[5] Shull GH (1914). Duplicate genes for capsule-form in Bursa bursa-pastoris. Z. Indukt. Abstamm. Vererbungsl.

[6] Tian F (2011). Genome-wide association study of leaf architecture in the maize nested association mapping population. Nat. Genet..

[7] Liedl BE, Anderson NO (1993). Reproductive barriers: identification, uses and circumvention. Plant Breed. Rev..

[8] Kumar S, Singh P (2005). Mechanisms for hybrid development in vegetables. J. N. Seeds.

[9] Nishi S (1967). F_1_ seed production in Japan. Proc. XVIII Int. Hort. Cong.

[10] da Silva Dias JC (2014). Guiding strategies for breeding vegetable cultivars. Agric. Sci..

[11] Hayes HK, Jones D (1917). First generation crosses in cucumbers. Conn. Storrs. Agric. Exp. Stn. Res. Rpt..

[12] Daunay, M. C. *Eggplant in Vegetables II* 163–220 (Springer, 2008).

[13] Singh, P. K., Dasgupta, S. K. & Tripathi, S. K. *Hybrid Vegetable Development* (CRC Press, 2005).

[14] Colombo N, Galmarini CR (2017). The use of genetic, manual and chemical methods to control pollination in vegetable hybrid seed production: a review. Plant Breed..

[15] Krieger U, Lippman ZB, Zamir D (2010). The flowering gene *SINGLE FLOWER TRUSS* drives heterosis for yield in tomato. Nat. Genet..

[16] Zhang WL (2020). Cloning and sequence analysis of *CsMYB108* gene in cucumber (*Cucumis sativa* L.). Mol..

[17] Liu T (2020). Enhanced photosynthetic activity in pak choi hybrids is associated with increased grana thylakoids in chloroplasts. Plant J..

[18] Kakizaki Y (1931). Hybrid vigor in egg-plants and its practical utilization. Genetics.

[19] Balwani A, Patel J, Acharya R, Gohil D, Dhruve J (2017). Heterosis for fruit yield and its component traits in brinjal (*Solanum melongena* L.). J. Pharmacogn. Phytochem..

[20] Makani A, Patel A, Bhatt M, Patel P (2013). Heterosis for yield and its contributing attributes in brinjal (*Solanum melongena* L.). bioscan.

[21] Tamta S, Singh J (2018). Heterosis in tomato for growth and yield traits. Int. J. Veg. Sci..

[22] Spaldon S, Hussain S, Jabeen N, Lay P (2015). Heterosis studies for earliness, fruit yield and yield attributing traits in chilli (*Capsicum Annum* L.). Bioscan.

[23] Kong X (2020). Transcriptome analysis of biological pathways associated with heterosis in Chinese cabbage. Genomics.

[24] Li H (2018). Transcriptome and DNA methylome reveal insights into yield heterosis in the curds of broccoli (*Brassica oleracea* L. var. *italic*). BMC Plant Biol..

[25] Sharma M, Singh Y, Singh SK, Dhangrah V (2016). Exploitation of gynoecious lines in cucumber (*Cucumis Sativus* L.) for heterosis breeding. Int. J. Bio-Resour. Stress Manag..

[26] El-Adl A, Abd El-Hadi A, Fathy HM, Abdein M (2014). Heterosis, Heritability and Combining Abilities for some Earliness Traits in Squash (*Cucurbita pepo*, L.). Alex. Sci. Exch. J..

[27] Liu J, Li M, Zhang Q, Wei X, Huang X (2020). Exploring the molecular basis of heterosis for plant breeding. J. Integr. Plant Biol..

[28] Qin J (2013). Identification and characterization of a repertoire of genes differentially expressed in developing top ear shoots between a superior hybrid and its parental inbreds in Zea mays L. Mol. Genet. Genom..

[29] Kumar R, Kumar S, Kumar D, Kansal S (2018). Heterosis, combining ability and gene action studies for insect-pest and disease resistance in cucumber. Electron. J. Plant Breed..

[30] Kumar A, Sharma V, Jain BT, Kaushik P (2020). Heterosis breeding in eggplant (*Solanum melongena* L.): Gains and provocations. Plants.

[31] Fujimoto, R. et al. Recent research on the mechanism of heterosis is important for crop and vegetable breeding systems. *Breed. Sci*. **68**, 145–158 (2018). 10.1270/jsbbs.17155 PMC5982191 29875598

[32] Shang LG, Gao ZY, Qian Q (2017). Progress in Understanding the Genetic Basis of Heterosis in Crops. Chin. Bull. Bot..

[33] Herath, H. N., Rafii, M., Ismail, S., Nakasha, J. J. & Ramlee, S. Improvement of important economic traits in chilli through heterosis breeding: a review. *J. Hortic. Sci. Biotechnol.* **96**, 1–10 (2020).

[34] Mallikarjunarao K, Badu M, Bandi HRK, Tripathy B (2020). Heterosis breeding in Bitter Gourd (*Momordica charantia* L.): a review. J. Pharmacogn. Phytochem..

[35] Davenport CB (1908). Degeneration, albinism and inbreeding. Science.

[36] Bruce A (1910). The Mendelian theory of heredity and the augmentation of vigor. Science.

[37] Jones DF (1917). Dominance of linked factors as a means of accounting for heterosis. Genetics.

[38] East EM (1936). Heterosis. Genetics.

[39] Dranginis A (1990). Binding of yeast al and α2 as a heterodimer to the operator DNA of a haploid-specific gene. Nature.

[40] Hollick JB, Chandler VL (1998). Epigenetic allelic states of a maize transcriptional regulatory locus exhibit overdominant gene action. Genetics.

[41] Stuber CW, Lincoln SE, Wolff D, Helentjaris T, Lander E (1992). Identification of genetic factors contributing to heterosis in a hybrid from two elite maize inbred lines using molecular markers. Genetics.

[42] Schnable, P. S. & Springer, N. M. Progress toward understanding heterosis in crop plants. *Annu. Rev. Plant Biol*. **64**, 71–88 (2013). 10.1146/annurev-arplant-042110-103827 23394499

[43] Wright, S. *Evolution and the Genetics of Populations, volume 1: Genetic and Biometric Foundations* (University of Chicago press, 1984).

[44] Sheridan, A. Cross breeding and heterosis Animal Breeding. Anim. Breed. Abst. **49**, 131–144 (1981).

[45] Sao A, Mehta N (2010). Heterosis in relation to combining ability for yield and quality attributes in brinjal (*Solanum melongena* L.). Electron. J. Plant Breed..

[46] Yu S (1997). Importance of epistasis as the genetic basis of heterosis in an elite rice hybrid. P. Natl Acad. Sci. USA..

[47] Zhong JC (1994). Active gene effect hypothesis. J. Southwest Nationalities Nat. Sci. Ed..

[48] Kermicle J (1970). Dependence of the R-mottled aleurone phenotype in maize on mode of sexual transmission. Genetics.

[49] Zhang M (2014). Genome-wide high resolution parental-specific DNA and histone methylation maps uncover patterns of imprinting regulation in maize. Genome Res..

[50] Bao WK (1990). Opportunity and risk–considerations for 40 years of breeding research. Plant J..

[51] Goff SA (2011). A unifying theory for general multigenic heterosis: energy efficiency, protein metabolism, and implications for molecular breeding. N. Phytol..

[52] Yao H, Gray AD, Auger DL, Birchler JA (2013). Genomic dosage effects on heterosis in triploid maize. Proc. Natl Acad. Sci. USA..

[53] Fu D (2014). Utilization of crop heterosis: a review. Euphytica.

[54] Chen ZJ (2010). Molecular mechanisms of polyploidy and hybrid vigor. Trends Plant Sci..

[55] Birchler JA, Yao H, Chudalayandi S, Vaiman D, Veitia RA (2010). Heterosis. Plant Cell.

[56] Liu C, Wang M, Wang L, Guo Q, Liang G (2018). Extensive genetic and DNA methylation variation contribute to heterosis in triploid loquat hybrids. Genome.

[57] Zhao X, Chai Y, Liu B (2007). Epigenetic inheritance and variation of DNA methylation level and pattern in maize intra-specific hybrids. Plant Sci. (Amst., Neth.).

[58] He G (2010). Global epigenetic and transcriptional trends among two rice subspecies and their reciprocal hybrids. Plant Cell.

[59] Shen H (2012). Genome-wide analysis of DNA methylation and gene expression changes in two Arabidopsis ecotypes and their reciprocal hybrids. Plant Cell.

[60] Lauss K (2018). Parental DNA methylation states are associated with heterosis in epigenetic hybrids. Plant Physiol..

[61] Shivaprasad PV, Dunn RM, Santos BA, Bassett A, Baulcombe DC (2012). Extraordinary transgressive phenotypes of hybrid tomato are influenced by epigenetics and small silencing RNAs. EMBO J..

[62] Cerruti, E. et al. Epigenetic bases of grafting-induced vigour in eggplant. *bioRxiv*10.1101/831719 (2019).

[63] Kenchanmane, R. S. K. & Niederhuth, C. E. Epigenetic diversity and application to breeding. Adv. Bot. Res. **88**, 49–86 (2018).

[64] Gouil Q, Novák O, Baulcombe DC (2016). *SLTAB2* is the paramutated *SULFUREA* locus in tomato. J. Exp. Bot..

[65] Hagemann RSomatische (1969). Konversion (Paramutation) am sulfurea Locus von *Lycopersicon esculentum* Mill. Theor. Appl. Genet..

[66] Law JA, Jacobsen SE (2010). Establishing, maintaining and modifying DNA methylation patterns in plants and animals. Nat. Rev. Genet..

[67] Kundariya H (2020). MSH1-induced heritable enhanced growth vigor through grafting is associated with the RdDM pathway in plants. Nat. Commun..

[68] Barber WT (2012). Repeat associated small RNAs vary among parents and following hybridization in maize. Proc. Natl Acad. Sci. USA..

[69] Nogueira, F. T. Tomato epigenetics: deciphering the “beyond” genetic information in a vegetable fleshy-fruited crop in Epigenetics in Plants of Agronomic Importance: Fundamentals and Applications 247–265 (Springer, 2019).

[70] Molnar A (2010). Small silencing RNAs in plants are mobile and direct epigenetic modification in recipient cells. Science.

[71] Lippman Z (2004). Role of transposable elements in heterochromatin and epigenetic control. Nature.

[72] Vance V, Vaucheret H (2001). RNA silencing in plants-defense and counterdefense. Science.

[73] Jenuwein T, Allis CD (2001). Translating the histone code. Science.

[74] Huang X, Han B (2014). Natural variations and genome-wide association studies in crop plants. Annu. Rev. Plant Biol..

[75] Huang X (2016). Genomic architecture of heterosis for yield traits in rice. Nature.

[76] Lippman ZB, Zamir D (2007). Heterosis: revisiting the magic. Trends Genet..

[77] Xiao J, Li J, Yuan L, Tanksley SD (1995). Dominance is the major genetic basis of heterosis in rice as revealed by QTL analysis using molecular markers. Genetics.

[78] Groszmann M (2015). Hormone-regulated defense and stress response networks contribute to heterosis in Arabidopsis F1 hybrids. Proc. Natl Acad. Sci. USA..

[79] Song GS (2010). Comparative transcriptional profiling and preliminary study on heterosis mechanism of super-hybrid rice. Mol. Plant.

[80] Chen ZJ (2013). Genomic and epigenetic insights into the molecular bases of heterosis. Nat. Rev. Genet..

[81] Ni Z (2009). Altered circadian rhythms regulate growth vigour in hybrids and allopolyploids. Nature.

[82] Baranwal VK, Mikkilineni V, Zehr UB, Tyagi AK, Kapoor S (2012). Heterosis: emerging ideas about hybrid vigour. J. Exp. Bot..

[83] Chen, S. R. *Vegetable Breeding* (Agricultural Press, 1980).

[84] Preuss SB (2008). Multimegabase silencing in nucleolar dominance involves siRNA-directed DNA methylation and specific methylcytosine-binding proteins. Mol. Cell.

[85] Wang L (2015). Hybrid mimics and hybrid vigor in Arabidopsis. Proc. Natl Acad. Sci. USA..

[86] Paschold A (2012). Complementation contributes to transcriptome complexity in maize (*Zea mays* L.) hybrids relative to their inbred parents. Genome Res..

[87] Li D (2016). Integrated analysis of phenome, genome, and transcriptome of hybrid rice uncovered multiple heterosis-related loci for yield increase. Proc. Natl Acad. Sci. USA..

[88] Springer NM, Stupar RM (2007). Allelic variation and heterosis in maize: how do two halves make more than a whole?. Genome Res..

[89] Barboza, G. E. et al. Solanaceae in Flowering plants. Eudicots 295–357 (Springer, 2016).

[90] Khush GS (2000). Rice germplasm enhancement at IRRI. Phillipp. J. Crop Sci..

[91] Donald CT (1968). The breeding of crop ideotypes. Euphytica.

[92] Yeager A (1927). Determinate growth in the tomato. J. Hered..

[93] Jiang K, Liberatore KL, Park SJ, Alvarez JP, Lippman ZB (2013). Tomato yield heterosis is triggered by a dosage sensitivity of the florigen pathway that fine-tunes shoot architecture. PLoS Genet..

[94] Khan A, Jindal S (2016). Exploiting yield potential in tomato (*Solanum lycopersicum* L.) through heterosis breeding. Plant Gene Trait.

[95] Wellington, R. *Influence of Crossing in Increasing the Yield of the Tomato* (New York Agricultural Experiment Station, 1912).

[96] Tschermak, E. V. Steigerung der Ertragsfähigkeit der Tomaten durch Bastardierung in der F_1_ Generation. *Nachr. Dtseh. Landwirtsch. Ges. Öst*. **51** (1918).

[97] Laterrot H (1973). Résistance de la tomate au virus de la mosaïque du tabac. Difficultés rencontrées pour la sélection de variétés résistantes. Ann. Am.élior Plantes.

[98] Soost R (1959). Tobacco mosaic resistance. TGC Rep..

[99] Lapushner, D. & Frankel, R. Rationale and practice of tomato F_1_ hybrid breeding and seed production. *Monographic di Genetica Agraria IV. Roma* 259–273 (1979).

[100] Philouze, J. Les hybrides de la tomate: leur intérêt, les techniques d’hybridation, l’utilisation de la stérilité mâle. *PHM Revue Hortieole.* **164**, 11–18 (1976).

[101] Naresh BV, Dubey A, Tiwari P, Dabbas M (2014). Line x Tester analysis for yield components and cercospora leaf spot resistance in brinjal (*Solanum melongena* L.). Electron. J. Plant Breed..

[102] Pandey S, Singh B, Singh M, Rai M (2005). Heterosis in cucumber (*Cucumis sativus* L.). Veg. Sci..

[103] Xu Y (2012). Whole-genome strategies for marker-assisted plant breeding. Mol. Breed..

[104] Xing L (2018). Heterotic group classification of 63 inbred lines and hybrid purity identification by using SSR markers in winter cabbage (*Brassica oleracea* L. var. *capitata*). Hortic. Plant J..

[105] Muthuselvi R, Praneetha S (2019). Molecular basis of self-incompatibility in vegetable crops. Int. J. Chem. Stud..

[106] Earle, E., Stephenson, C., Walters, T. & Dickson, M. Cold-tolerant Ogura CMS brassica vegetables for horticultural use. *Cruciferae Newsletter* **16**, 80–81 (1994).

[107] Pelletier G (1983). Intergeneric cytoplasmic hybridization in Cruciferae by protoplast fusion. Mol. Gen. Genet. MGG.

[108] Xiao Z (2019). Overcoming cabbage crossing incompatibility by the development and application of self-compatibility-QTL-specific markers and genome-wide background analysis. Front. Plant Sci..

[109] Atanassova, B. & Georgiev, H. Using genic male sterility in improving hybrid seed production in tomato (*Lycopersicon esculentum* Mill.). *Acta Hortic* **579**, 185–188 (2000).

[110] Dhall, R. Status of male sterility in vegetables for hybrid development. A review. *Adv. Hortic. Sci.* **24**, 263–279 (2010).

[111] Georgiev, H. Heterosis in tomato breeding in Genetic improvement of tomato 83–98 (Springer, 1991).

[112] Mizanur, M., Khan, R. & Isshiki, S. Cytoplasmic male sterility in eggplant. *Hortic. J*. **85**, 1–7 (2016).

[113] Krommydas KS (2016). Development and fertility restoration of CMS eggplant lines carrying the cytoplasm of Solanum violaceum. J. Agric. Sci..

[114] Lin SW (2015). Restorer breeding in sweet pepper: introgressing Rf allele from hot pepper through marker-assisted backcrossing. Sci. Hortic..

[115] Liu C, Ma N, Wang PY, Fu N, Shen HL (2013). Transcriptome sequencing and de novo analysis of a cytoplasmic male sterile line and its near-isogenic restorer line in chili pepper (*Capsicum annuum* L.). PLoS ONE.

[116] Kumar S (2013). Male sterility in vegetables. Olericulture Fundam. Veg. Prod..

[117] Du, M. et al. A biotechnology-based male-sterility system for hybrid seed production in tomato. *Plant J*. **102**, 1090–1100 (2020). 10.1111/tpj.14678 PMC7317546 31923323

[118] Papadopoulou E, Grumet R (2005). Brassinosteriod-induced femaleness in cucumber and relationship to ethylene production. HortScience.

[119] Shannon S, Guardia deLa (1969). M. D. Sex expression and the production of ethylene induced by auxin in the cucumber (*Cucumis sativum* L.). Nature.

[120] Melchinger AE, Gumber RK (1998). Overview of heterosis and heterotic groups in agronomic crops. Concepts Breed. Heterosis Crop Plants.

[121] Chen J (2019). Genome‐wide association analyses reveal the genetic basis of combining ability in rice. Plant Biotechnol. J..

[122] Mather K (1942). The balance of polygenic combinations. J. Genet..

[123] Griffing B (1956). Concept of general and specific combining ability in relation to diallel crossing systems. Aust. J. Biol. Sci..

[124] Toker, C. et al. Association between heterosis and genetic distance based on morphological traits and SSR markers in Cicer species. In *Pre Breeding–fishing in the Gene Pool»*. *Abstract of Oral Presentation And Posters of the European Plant Genetic Resources Conference* 85 (2013).

[125] Jagosz B (2011). The relationship between heterosis and genetic distances based on RAPD and AFLP markers in carrot. Plant Breed..

[126] Sharma BB, Sharma VK, Dhakar MK, Punetha S (2013). Combining ability and gene action studies for horticultural traits in garden pea: A review. Afr. J. Agric. Res..

[127] Fasahat P, Rajabi A, Rad J, Derera J (2016). Principles and utilization of combining ability in plant breeding. Biom. Biostat. Int J..

[128] Rajendrakumar, P., Hariprasanna, K. & Seetharama, N. Prediction of heterosis in crop plants—status and prospects. *J. Exp. Agric. Int.* **9**, 1–16 (2015).

[129] Singh A, Pan R, Bhavana P (2013). Heterosis and combining ability analysis in bittergourd (*Momordica charantia* L.). Bioscan.

[130] Kenga R, Alabi S, Gupta S (2004). Combining ability studies in tropical sorghum (*Sorghum bicolor* (L.) *Moench*). Field Crops Res..

[131] Franco MC (2001). Combining ability for nodulation in common bean (*Phaseolus vulgaris* L.) genotypes from Andean and Middle American gene pools. Euphytica.

[132] Gerdes JT, Tracy WF (1993). Pedigree diversity within the Lancaster surecrop heterotic group of maize. Crop Sci..

[133] Arunachalam, V. & Bandyopadhyay, A. Limits to genetic divergence for occurrence of heterosis Experimental evidence from crop plants. *Indian J. Genet. Plant Breed. (India)* **44**, 548–554 (1984).

[134] Zhong-hu H (1991). An investigation of the relationship between the F 1 potential and the measures of genetic distance among wheat lines. Euphytica.

[135] Kaeppler, S. Heterosis: many genes, many mechanisms—end the search for an undiscovered unifying theory. *Int. Sch. Res. Not.* **2012**, 1–12 (2012).

[136] Poehlman, J. & Sleper, D. *Breeding Field Crops* (Iowa State University Press, 1995).

[137] Tomkowiak A, Bocianowski J, Kwiatek M, Kowalczewski P (2020). Ł. Dependence of the heterosis effect on genetic distance, determined using various molecular markers. Open Life Sci..

[138] Xiao J, Li J, Yuan L, McCouch S, Tanksley S (1996). Genetic diversity and its relationship to hybrid performance and heterosis in rice as revealed by PCR-based markers. Theor. Appl. Genet..

[139] Geleta L, Labuschagne M, Viljoen C (2004). Relationship between heterosis and genetic distance based on morphological traits and AFLP markers in pepper. Plant Breed..

[140] José MA, Iban E, Silvia A, Pere A (2005). Inheritance mode of fruit traits in melon: Heterosis for fruit shape and its correlation with genetic distance. Euphytica.

[141] Xu S, Xu Y, Gong L, Zhang Q (2016). Metabolomic prediction of yield in hybrid rice. Plant J..

[142] Bo K (2019). QTL mapping and genome-wide association study reveal two novel loci associated with green flesh color in cucumber. BMC Plant Biol..

[143] WEI, S. et al. Evaluation and Genome-wide Association Study (GWAS) of seedling thermotolerance in cucumber core germplasm. *J. Plant Genet. Resour.* **23**, 1223–1231 (2019).

[144] Huang, X. et al. Genomic analysis of hybrid rice varieties reveals numerous superior alleles that contribute to heterosis. *Nat. Commun.* **6**, 1–9 (2015). 10.1038/ncomms7258 PMC4327311 25651972

[145] Frascaroli, E. et al. Classical genetic and quantitative trait loci analyses of heterosis in a maize hybrid between two elite inbred lines. *Genetics* **176**, 625–644 (2007). 10.1534/genetics.106.064493 PMC1893040 17339211

[146] Guo T (2014). Genetic basis of grain yield heterosis in an “immortalized F 2” maize population. Theor. Appl. Genet..

[147] Alpert K, Grandillo S, Tanksley S (1995). fw 2.2: a major QTL controlling fruit weight is common to both red-and green-fruited tomato species. Theor. Appl. Genet..

[148] Zhuang J, Fan Y, Wu J, Xia Y, Zheng K (2000). Identification of over-dominance QTL in hybrid rice combinations. Hereditas.

[149] Mei H (2005). Gene actions of QTLs affecting several agronomic traits resolved in a recombinant inbred rice population and two backcross populations. Theor. Appl. Genet..

[150] Li L (2008). Dominance, overdominance and epistasis condition the heterosis in two heterotic rice hybrids. Genetics.

[151] Jiang G, Zeng J, He Y (2014). Analysis of quantitative trait loci affecting chlorophyll content of rice leaves in a double haploid population and two backcross populations. Gene.

[152] Wei X (2015). Heterotic loci for various morphological traits of maize detected using a single segment substitution lines test-cross population. Mol. Breed..

[153] Li, H. et al. Quantitative trait locus analysis of heterosis for plant height and ear height in an elite maize hybrid zhengdan 958 by design III. *BMC Genom.* **18**, 36 (2017). 10.1186/s12863-017-0503-9 PMC5392948 28415964

[154] Guo X (2013). Mapping heterotic loci for yield and agronomic traits using chromosome segment introgression lines in cotton. J. Integr. Plant Biol..

[155] Tian S (2019). Overdominance is the major genetic basis of lint yield heterosis in interspecific hybrids between G. hirsutum and G. barbadense. Heredity.

[156] Yang M (2017). Genomic architecture of biomass heterosis in Arabidopsis. Proc. Natl Acad. Sci. USA..

[157] DeVicente M, Tanksley S (1993). QTL analysis of transgressive segregation in an interspecific tomato cross. Genetics.

[158] Zhou G (2012). Genetic composition of yield heterosis in an elite rice hybrid. Proc. Natl Acad. Sci. USA..

[159] Radoev, M., Becker, H. C. & Ecke, W. J. G. Genetic analysis of heterosis for yield and yield components in rapeseed (*Brassica napus* L.) by quantitative trait locus mapping. *Genetics* **179**, 1547–1558 (2008). 10.1534/genetics.108.089680 PMC2475754 18562665

[160] Li C (2018). Genetic basis of heterosis for yield and yield components explored by QTL mapping across four genetic populations in upland cotton. BMC Genom..

[161] Luo X (2009). Additive and over‐dominant effects resulting from epistatic loci are the primary genetic basis of heterosis in rice. J. Integr. Plant Biol..

[162] Tang J (2010). Dissection of the genetic basis of heterosis in an elite maize hybrid by QTL mapping in an immortalized F_2_ population. Theor. Appl. Genet..

[163] Liu, H. et al. Genome‐wide identification and analysis of heterotic loci in three maize hybrids. *Plant Biotechnol. J.* **18**, 185–194 (2020). 10.1111/pbi.13186 PMC6920156 31199059

[164] Ma L, Wang Y, Ijaz B, Hua J (2019). Cumulative and different genetic effects contributed to yield heterosis using maternal and paternal backcross populations in Upland cotton. Sci. Rep. UK..

[165] Li Z-K (2001). Overdominant epistatic loci are the primary genetic basis of inbreeding depression and heterosis in rice. I. Biomass. Grain Yield..

[166] Luo L (2001). Overdominant epistatic loci are the primary genetic basis of inbreeding depression and heterosis in rice. II. Grain Yield Compon..

[167] Zhu D, Zhou G, Xu C, Zhang Q (2016). Genetic components of heterosis for seedling traits in an elite rice hybrid analyzed using an immortalized F_2_ population. J. Genet. Genom..

[168] Song F (2011). Heterosis for plant height and ear position in maize revealed by quantitative trait loci analysis with triple testcross design. Acta Agronomica Sin..

[169] Liang, Q., Shang, L., Wang, Y. & Hua, J. Partial dominance, overdominance and epistasis as the genetic basis of heterosis in upland cotton (*Gossypium hirsutum* L.). *PLoS ONE* **10** (2015). 10.1371/journal.pone.0143548 PMC4664285 26618635

[170] Liu R, Wang B, Guo W, Wang L, Zhang T (2011). Differential gene expression and associated QTL mapping for cotton yield based on a cDNA-AFLP transcriptome map in an immortalized F 2. Theor. Appl. Genet..

[171] Semel Y (2006). Overdominant quantitative trait loci for yield and fitness in tomato. P. Natl Acad. Sci. USA..

[172] Larièpe A (2012). The genetic basis of heterosis: multiparental quantitative trait loci mapping reveals contrasted levels of apparent overdominance among traits of agronomical interest in maize (*Zea mays* L.). Genetics.

[173] Li, Q. et al. The identification of Cucumis sativus *Glabrous 1* (*CsGL1*) required for the formation of trichomes uncovers a novel function for the homeodomain-leucine zipper I gene. *J. Exp. Bot.* **66**, 2515–2526 (2015). 10.1093/jxb/erv046 25740926

[174] Hua, J. et al. Single-locus heterotic effects and dominance by dominance interactions can adequately explain the genetic basis of heterosis in an elite rice hybrid. *P. Natl Acad. Sci. USA.* **100**, 2574–2579 (2003). 10.1073/pnas.0437907100 PMC151382 12604771

[175] Zhang L (2017). A natural tandem array alleviates epigenetic repression of *IPA1* and leads to superior yielding rice. Nat. Commun..

[176] Yu Y (2020). Genome sequence and QTL analyses using backcross recombinant inbred lines (BILs) and BILF_1_ lines uncover multiple heterosis-related loci. Int. J. Mol. Sci..

[177] Liu Y (2020). Identification of quantitative trait loci for kernel-related traits and the heterosis for these traits in maize (*Zea mays* L.). Mol. Genet. Genom..

[178] Xu M (2020). Heterotic loci analysis for root traits of maize seedlings using an SSSL test population under different nitrogen conditions. Mol. Breed..

[179] Li X (2020). Maize *GOLDEN2-LIKE* genes enhance biomass and grain yields in rice by improving photosynthesis and reducing photoinhibition. Commun. Biol..

[180] Mohapatra PP, Batra V (2017). Tissue culture of potato (*Solanum tuberosum* L.): A review. Int. J. Curr. Microbiol. Appl. Sci..

[181] Khanday I, Skinner D, Yang B, Mercier R, Sundaresan V (2019). A male-expressed rice embryogenic trigger redirected for asexual propagation through seeds. Nature.

[182] Wang C (2019). Clonal seeds from hybrid rice by simultaneous genome engineering of meiosis and fertilization genes. Nat. Biotechnol..

[183] Williams W (1959). The isolation of ‘pure lines’ from F_1_ hybrids of tomato, and the problem of heterosis in inbreeding crop species. J. Agric. Sci..

[184] Sarawat P, Stoddard F, Marshall D (1993). Derivation of superior F_5_ lines from heterotic hybrids in pea. Euphytica.

[185] Manrique-Carpintero NC (2018). Genome reduction in tetraploid potato reveals genetic load, haplotype variation, and loci associated with agronomic traits. Front. plant Sci..

[186] Wang X (2011). The genome of the mesopolyploid crop species Brassica rapa. Nat. Genet..

[187] Li, X. et al. Establishment of Agrobacterium-mediated genetic transformation and application of CRISPR/Cas9 gene-editing system to Chinese cabbage (*Brassica rapa* L. ssp. *pekinensis*). (2020).

[188] Liu S (2014). The Brassica oleracea genome reveals the asymmetrical evolution of polyploid genomes. Nat. Commun..

[189] Ma C (2019). Efficient BoPDS gene editing in cabbage by the CRISPR/Cas9 system. Hortic. Plant J..

[190] Kitashiba H (2014). Draft sequences of the radish (*Raphanus sativus* L.) genome. DNA Res..

[191] Kim K (2020). Quantitative Trait Loci (QTLs) Associated with Microspore Culture in Raphanus sativus L.(Radish). Genes.

[192] Consortium TG (2012). The tomato genome sequence provides insights into fleshy fruit evolution. Nature.

[193] Lee MH (2020). Temporal and spatial expression analysis of shoot-regeneration regulatory genes during the adventitious shoot formation in hypocotyl and cotyledon explants of tomato (cv. Micro-Tom). Int. J. Mol. Sci..

[194] Consortium PGS (2011). Genome sequence and analysis of the tuber crop potato. Nature.

[195] Zhang, C., Wang, D., Yang, Y. & Chen, Q. Construction of Potato DM1-3-516-R44 (DM) transgenic system based on agrobacterium transformation. (2020).

[196] Kim S (2014). Genome sequence of the hot pepper provides insights into the evolution of pungency in Capsicum species. Nat. Genet..

[197] Qin C (2014). Whole-genome sequencing of cultivated and wild peppers provides insights into Capsicum domestication and specialization. Proc. Natl Acad. Sci. USA..

[198] Chee MJY, Lycett GW, Chin CF (2018). Development of a direct transformation method by GFP screening and in vitro whole plant regeneration of *Capsicum frutescens* L. Electron. J. Biotechnol..

[199] Hirakawa H (2014). Draft genome sequence of eggplant (*Solanum melongena* L.): the representative solanum species indigenous to the old world. DNA Res..

[200] Khan, H. An Efficient Plant Regeneration System Via Leaf Derived Callus of *Solanum melongena* L in Propagation and Genetic Manipulation of Plants 93-100 (Springer, 2021).

[201] Huang S (2009). The genome of the cucumber, *Cucumis sativus* L. Nat. Genet..

[202] Du, C., Fan, H., Liu, C. & Si, Y. Improved the Agrobacterium tumefaciens-mediated transformation of cucumber by a modified the using of antibiotics and acetosyringone. (2020).

[203] Garcia-Mas J (2012). The genome of melon (*Cucumis melo* L.). Proc. Natl Acad. Sci. USA..

[204] Chenarani Z, Shokouhifar F, Mamarabadi M (2018). Study the Effects of Explants and Hormonal Levels onDirect Regeneration in Melon (*Cucumis melo* L., cv. *Khatooni*). J. Hortic. Sci..

[205] Guo B (2013). Comparative proteomic analysis of embryos between a maize hybrid and its parental lines during early stages of seed germination. PLoS ONE.

[206] Vasudevan V, Siva R, Krishnan V, Manickavasagam M (2020). Polyamines, sonication and vacuum infiltration enhances the Agrobacterium-mediated transformation in watermelon (*Citrullus lanatus* Thunb.). S. Afr. J. Bot..

[207] Montero‐Pau J (2018). De novo assembly of the zucchini genome reveals a whole‐genome duplication associated with the origin of the Cucurbita genus. Plant Biotechnol. J..

[208] Sun H (2017). Karyotype stability and unbiased fractionation in the paleo-allotetraploid Cucurbita genomes. Mol. Plant.

[209] Guo J, Li Y, He C, Yan Y, Yu X (2019). Establishing a High-efficiency Regeneration System in Pumpkin (Cucurbita moschata). Chin. Bull. Bot..

