# Supplementary material for: Molecular basis of heterosis and related breeding strategies reveal its importance in vegetable breeding
Source: Hortic Res. 2021 Jun 1;8:120. doi: 10.1038/s41438-021-00552-9 (PMC8166827; doi:10.1038/s41438-021-00552-9)
Supplement: Supplementary file 1 — Supplymental Table 1 [file 41438_2021_552_MOESM1_ESM.pdf]

Supplymental Table S1 QTL effects of heterosis in crops and vegetables

Quantitative trait locus effect	Genetical population	Species	Reference
Dominance	RIL, 2BC	*O.sativa*	Xiao et al. (1995)[77]
	1495 hybrids	*O.sativa*	Huang et al. (2015)[78]
	RIL, 3TC	*Zea mays*	Frascaroli et al. (2007)[79]
	IF_2	*Z.mays*	Guo et al. (2014)[80]
Partial dominance	Ils, F_2, BC_1	*S.lycopersicum*	Alpert et al. (1995)[81]
Overdominance	F_2	*O.sativa*	Zhuang et al. (2000)[82]
	RIL, 2BC	*O.sativa*	Mei et al. (2005)[83]
	RIL, 2BC	*O.sativa*	Li et al. (2008)[84]
	DH, 2BC	*O.sativa*	Jiang et al. (2014)[85]
	SSSLs, TC	*Z.mays*	Wei et al. (2015)[86]
	RIL	*Z.mays*	Li et al. (2017)[87]
	CSILs, TC	*G.hirsutum*	Guo et al. (2013)[88]
	ILs	*G.hirsutum*	Tian et al. (2019)[89]
	ILs, TC	*S.lycopersicum*	Semel et al. (2006)[90]
Pseudo–overdominance	3RIL, 9TC	*Z.mays*	Larièpe et al. (2012)[91]
	RIL	*S.vulgare*	Li et al. (2015)[92]
Epistasis	IF_2	*O.sativa*	Hua et al. (2003)[93]
Dominance, overdominance	RIL	*O.sativa*	Zhang et al. (2017)[94]
	BILs	*O.sativa*	Yu et al. (2020)[95]
Dominance, epistasis	RIL, IF_2	*Z.mays*	Liu et al. (2020c)[96]
	IF_2	*Z.mays*	Tang et al. (2010)[97]
Partial dominance, overdominance	$14F_2$ populations	*O.sativa*	Huang et al. (2016)[75]
	5360 hybrids	*Z.mays*	Liu et al. (2020a)[98]
	2BC	*G.hirsutum*	Ma et al. (2019)[99]
Overdominance, epistasis	$F_{2:3}$	*O.sativa*	Yu et al. (1997)[46]
	RIL, 2BC, 2TC	*O.sativa*	Li et al. (2001)[100]
	RIL, 2BC, 2TC	*O.sativa*	Luo et al. (2001)[101]
	IF_2	*O.sativa*	Zhu et al. (2016)[102]
	RIL, 3TC	*Z.mays*	Song et al. (2011)[103]
Partial dominance, overdominance, epistasis	$F_{2:3}$	*G.hirsutum*	Liang et al. (2015)[104]
Dominance, overdominance, epistasis	2RIL, 4BC	*O.sativa*	Li et al. (2008)[105]
	IF_2	*O.sativa*	Zhou et al. (2012)[106]
	DH, TC	*B.campestris*	Radoev et al. (2008)[107]
Additive, partial dominance, overdominance	IF_2, $2BCF_1$	*G.hirsutum*	Li et al. (2018)[108]
Additive, overdominance, epistasis	RIL, 2BC	*O.sativa*	Luo et al. (2009)[109]
